# Developing and testing a South African Brief Cognitive Score in literate and illiterate people of mixed language groups

**DOI:** 10.4102/sajpsychiatry.v27i0.1327

**Published:** 2021-05-27

**Authors:** Clara M. Schutte, Mukhethwa Tsikane, Keorapetse Nchoe

**Affiliations:** 1 Department of Neurology, Faculty of Health Sciences, University of Pretoria, Pretoria, South Africa

**Keywords:** testing cognition, Folstein MMSE, SA Brief Cognitive Score, dementia, illiteracy

## Abstract

**Background:**

The Folstein mini mental state exam (MMSE) is the most commonly administered assessment of cognitive functioning; however, its utility in illiterate individuals is limited. In South Africa, more than eight million adults are considered functionally illiterate and cognitive evaluation using standard scales is inaccurate. Other countries have developed adapted MMSE scales for their local purposes.

**Aim:**

The first aim of this study was to develop a South African Brief Cognitive Score (SA BCS) for use in minimally literate or illiterate individuals. The second aim was to test this SA BCS against the original Folstein MMSE in patients with memory problems.

**Setting:**

The study was conducted in Tshilamba, Tshiombo, Tshifudi, Dzimauli and Pile in Venda as well as Rethabiseng and Zithobeni in Bronkhorstspruit for the illiterate study group, and Steve Biko Academic Hospital for the literate study groups.

**Methods:**

The SA BCS was developed considering our local requirements and substituting questions needing literacy with items that did not. Both the original Folstein MMSE and the SA BCS were administered to groups of literate and illiterate normal individuals. Thereafter, the tests were repeated in groups of literate and illiterate patients with cognitive decline.

**Results:**

Firstly, 33 illiterate and 31 literate subjects were assessed. The average original Folstein score was 29.29/30 for the literate subjects, and for the SA BCS 29.80. For the illiterate subjects, the average score for the original Folstein MMSE was 21.24/30 and for the SA BCS 27.45. Kruskall–Wallis equality of population rank test confirmed a significant improvement in the scores of the SA BCS in the illiterate group when compared to the original Folstein (*p* = 0.0001). In the second part of the study, 20 literate and 20 illiterate patients were assessed as before. In the literate group, the average original Folstein MMSE score was 20.5, while the average score for the South African BCS was 22.5. In the illiterate group, the average Folstein MMSE was 18.9; and the average score in the South African BCS was 22.8. The Kruskal–Wallis equality of population rank test showed a significant difference (*p* = 0.008) between the scores of the illiterate versus literate patients when the Folstein MMSE was used to assess cognition. With the SA BCS, no significant difference was found between the groups (*p* = 0.79).

**Conclusion:**

The SA BCS appears to have potential to be a more reliable scale when assessing cognition in illiterate or minimally literate subjects when compared to the original Folstein MMSE.

## Background and aim

The Folstein Mini Mental State Examination (MMSE) is a useful and well-known screening technique (a questionnaire) for cognitive impairment used worldwide to assess cognition of patients.^1^ The early identification of cognitive impairment is crucial for possible therapeutic intervention to reduce the burden on patients, family members and care givers and to lighten the socioeconomic burden on the country.

Being a brief, practical and user-friendly scale, the Folstein MMSE is considered one of the most popular methods for cognition evaluation. It provides global measurements of cognitive function based on 11 interview questions, which cover a broad range of cognitive domains, including orientation, registration, memory, attention, calculation, visuospatial functioning and praxis. A Folstein MMSE score of greater than or equal to 27/30 points indicates a normal cognition; below this, the score can indicate severe (< 9 points), moderate (10–18 points) or mild (19–26 points) cognitive impairment. The MMSE is effective as a screening technique to separate patients with cognitive impairment from those without. However, this method can not diagnose the cause for cognitive impairment and, hence, cannot replace a complete clinical assessment of mental status.^2^ In addition, the instrument relies heavily on verbal response and reading and writing – therefore, schooling can influence performance in this cognitive assessment test. The use of the MMSE might be inaccurate when evaluating illiterate individuals and those with a low level of education. It has been shown in several studies that these patients may perform poorly even when cognitively intact.^3,4,5,6,7^

In South Africa, an estimated population group of 2.9 to 4.2 million has never attended school and is considered illiterate. Furthermore, it is estimated that South Africa has between 7.4 and 8.5 million adults who are functionally illiterate. In contrast to illiterate persons (who are unable to read and write simple sentences in any language), functionally illiterate persons are regarded as adults with inadequate reading and writing skills to manage daily living and to do employment tasks requiring reading skills beyond a basic level.^8^

Observations made at the Steve Biko Academic Hospital Neurology clinic confirmed that many patients are either illiterate or minimally literate who do not perform well on the standard cognitive assessments used. In the case of the Folstein MMSE, some items on the questionnaire have to be omitted and, consequently, the value of using the MMSE in these patients is questionable and cognitive assessment is not accurate.

Countries such as China, Bangladesh and India have adapted the Folstein MMSE to accommodate their populations with different levels of education and cultural practices. In a study from China, researchers identified that the usual evaluation techniques for testing cognition required literacy as a prerequisite, which was impractical among elderly people born around World War II.^9^ At that time, mass education was less available for the general population, especially for women, peasants and people in rural areas. The Chinese adapted mental state examination (CAMSE) was then modified from the original MMSE by changing some items so as to minimise literacy dependency and to render them compatible with Chinese culture. The main structures were kept intact and no changes were made regarding scoring principles.^9^

Similar studies were done in Bangladesh where a modified MMSE version adapted to the cultural context of Bangladesh did not need education as precondition. The researchers tested the original MMSE and compared it to the Bangla-adapted MMSE (BAMSE) in illiterate and literate individuals, concluding that the BAMSE could be used as an instrument to assess cognitive function of normal elderly individuals irrespective of literacy skills.^10,11^ Another study from India aimed to develop a cognitive screening instrument for the illiterate rural elderly population of that country.^12^ The researchers changed the original MMSE to a Hindi-adapted MMSE (HSME) to assess cognition by modifying items that required reading and writing skills, showing that the Hindi MMSE was a useful tool for assessing the cognitive functioning of the tested elderly population.^12^

The aim of our study was therefore twofold: firstly, to develop a South African examination of mental state or cognition and compare it to the original Folstein MMSE in literate and illiterate healthy South African adults, and, secondly, to compare this South African Brief Cognitive Score (SA BCS) with the original Folstein MMSE in literate and illiterate patients with memory problems. Similar to the studies from other countries with a high illiteracy rate, this study will help investigate whether this SA BCS can be used as a cognitive screening technique among minimally literate and illiterate South African individuals as well as among patients with cognitive deterioration. With new treatment options becoming available, there is a need for the early recognition of cognitive impairment at the community and primary care level, and validated and simple tools are necessary to achieve this.^13^

## Methods part one

Development of the SA BCS and testing it against the original Folstein MMSE in healthy literate and illiterate participants

### Development of the SA Brief Cognitive Score

In the SA BCS, items that necessitate reading and writing were replaced by items that do not require an educational background or reading and writing skills. Selecting these items was achieved by consulting with colleagues who have been in practice for many years, as well as by comparing the different adapted versions of the MMSE in countries similar to South Africa.

In South Africa, there are individuals who struggle with the use of a calendar; therefore, the SA BCS incorporated questions on the time of the day (morning, afternoon or evening). However, it was assumed that most people would know the present year and month and therefore these items were kept in the questionnaire. Orientation to place was scored as per local settings; questions such as ‘which floor’ were eliminated because many rural places do not have buildings with floors. In the Hindi-adapted MMSE, the last question for place orientation was ‘which place is this/whose house is this’ – which may sometimes be open to different interpretations. Then we gave the scenario of spending money in decrements of 5; in the Hindi version, the researchers had used 20 minus 3 in a practical example of paying for a bus fare.^12^ For attention, we first asked subjects to say the days of the week backwards; this was based on the Hindi version of the MMSE, where the researchers had found this a good test.^12^ For registration/new learning, we used items that may be more well-known to a rural setting (mealie, fire, axe); this was also done in the Hindi MMSE, where subjects had to remember ‘mango, chair and coin’, rather than ‘apple, table, penny’.^12^ This was well received in our group of individuals.

Naming of objects was assessed by pointing to objects such as watch and button and asking the person to name them, and vernacular familiar tongue twister phrases were used to assess for repetition. The command was kept the same as in the original Folstein MMSE – this was also done in the Hindi MMSE version. A command that involved reading was substituted by asking the patient to copy the action in a picture of folded hands or by asking the person to copy the examiner’s folded hands. The Hindi version of the MMSE asked the patient to copy the examiner who closes his eyes, but this would then mean that there must be an additional person doing the test, since the examiner cannot see whether the subject is obeying the command.^12^ For sentence construction, we just asked the subject to verbalise how he/she would find out the interviewer’s name if they did not know this. The Hindi and Bangla versions of the MMSE used ‘tell me something about your house’ or the surroundings to prompt a sentence construction.^10,11,12^ Since many of our subjects had not frequently used a pencil or pen for drawing, we decided to substitute drawing with copying an easy construction of a figure (house) with matches and to lay a figure 8 with a string. This generated two sets of values eventually, termed SA BCS matches and SA BCS string. In the Hindi MMSE, subjects were asked to copy a figure of a square with a diamond within.^12^ Lastly, participants were asked to recall the three objects previously given.

The SA BCS was translated to vernacular by individuals who were not participating in the study, but were mother tongue speakers. Different individuals who were also not part of the study were then also asked to translate back from vernacular to English to assure the accuracy of the SA BCS.

### Study groups

The illiterate group consisted of a group of farmers from Tshilamba, Tshiombo, Tshifudi, Dzimauli and Pile in Venda as well as community members from Rethabiseng and Zithobeni in Bronkhorstspruit. The candidates who were interviewed were people who had never attended formal school, as well as subjects who had attended school up to a maximum of 7 years (Grade 5). After asking the local chieftain for permission, the registrar and her assistant (a master’s degree student) approached participants at their normal meeting points where they would congregate for farming workshops and activities.

The literate group is composed of volunteers who were staff members (clerks, nurses, electrophysiology staff, etc.) from Steve Biko Academic Hospital. All these candidates had at least matric-level qualifications.

### Subject evaluation

All individuals, literate and illiterate or minimally literate, were assessed with the two cognitive assessment techniques, namely the original Folstein MMSE and the SA BCS.

All participants were above the age 18 of years. Participants who had a previous history of psychiatric disorders, epilepsy, conditions that affect cognitive performance or known to be using illicit drugs were excluded from the study. The registrar doing the study, as well as the person assisting her, questioned the participants as to the presence or absence of the above.

The purpose of the study was explained to the subjects. All subjects were tested with the Folstein MMSE and then followed by the SA BCS or vice versa. The correct answers were not given to the subjects if they answered incorrectly. The interview was initially conducted on different days, first by testing them with the original Folstein MMSE, and after an interval of 5 weeks, they were tested with the SA BCS. This, however, unfortunately turned out to be very impractical, as many participants did not return for the second interview. Subsequently, both tests were done on the same day – in half of the subjects, the first test was conducted using the Folstein MMSE and in the other half, the interview was started using the SA BCS.

## Methods part two

Testing the SA BCS in literate and illiterate patients presenting to the Neurology Department with cognitive deterioration/memory problems.

### Study groups

All patients (literate: 20; illiterate/minimally literate: 20) aged 50–76 years seen in the Neurology Department at Steve Biko Academic Hospital with a history of memory problems or cognitive decline were included in the study. Patients with delirium or very advanced disease and thus unable to complete the test were excluded from the study. The registrar conducting the study assessed patients and excluded those with delirium or very advanced disease.

### Subject evaluation

The chosen patients were taken aside from the busy queue in the hospital to a quiet consulting room to let them rest and familiarise themselves with the hospital environment before the assessment was done. The purpose of the study was explained to the patients and their families, and consent from the patient and/or family was obtained. For one-half of the patients, the Folstein MMSE was conducted first, followed by a general and neurological examination. The South African BCS was conducted at the end of the consultation and vice versa for the other half of the patients. The idea was to account for possible practice effect by switching the sequence of testing around. We also did not provide the answers to the questions in the first test in an attempt to minimise the practice effect. The patients were tested in their home language by the registrar in the Department of Neurology. Both results were recorded in a data capturing sheet and compared.

### Ethical considerations

The study was approved by the Faculty of Health Sciences Research Ethics Committee of the University of Pretoria (reference numbers 8/2015 and 54/2018). Informed consent was obtained from all subjects or their family members before participating in the study.

## Results part one

There were 33 participants in the illiterate group and 31 in the literate group. Females predominated in both groups, with 23 females and 10 males in the literate group, and 26 females and 5 males in the literate group. Participants who had 0–2 years of schooling totalled 67%, and 33% had 3–5 years of schooling.

The average original Folstein score was 29.29/30 for the literate participants (range 26–30), and the scores for the SA BCS with matches and string were 29.80 and 29.77, respectively (ranges 28–30 in both). For the illiterate participants, the mean score for the original Folstein MMSE was 21.24/30 (range 11–28), and for the SA BCS with matches and string 27.45 and 27.48, respectively (range 21–30 in both) (see Table 1).

**TABLE 1:** Comparison of mini mental state exam and SA Brief Cognitive Score scores of the literate and illiterate participants.

Variable	Folstein MMSE		SA BCS matches		SA BCS string	
	Mean	range	Mean	range	Mean	range
Literate	29.29	26–30	29.80	28–30	29.77	28–30
Illiterate	21.24	11–28	27.45	21–30	27.48	21–30

MMSE, mini mental state exam; SA BCS, SA Brief Cognitive Score.

Statistical analysis showed that using the Kruskall–Wallis equality of population rank test, the scores of the SA BCS (string and match) both confirmed a significant improvement in the illiterate group when compared to the original Folstein (*p* = 0.0001) (*p* < 0.05 taken as significant). The illiterate participants thus performed significantly better with the SA BCS. Although the average scores improved slightly in the literate group, there was no statistical difference between the scores of the Folstein MMSE and the SA BCS in the literate participants.

## Results part two

During this part of the study, 40 patients were interviewed. There were 20 patients in each group (literate and illiterate). The literate group had a male predominance of 13, and the females were 7. The literate group consisted of 12 females and 8 males.

The patients were aged between 50 and 76 years. All patients underwent a thorough general and neurological examination carried out by the registrar who was doing the study. None of the patients had any focal neurological fallout on examination and treatable causes of cognitive impairment were excluded. In the literate group, the average original Folstein MMSE score was 20.5, with a mean of 18.9 (range 10–23), while the average score for the SA BCS was 22.5 with a mean of 22.8 (range 11–25). Since the SA BCS of the first part of the study showed that the results of the construction test using matches or a string were very similar (slightly better with the string), it was decided in this group of patients to only report the SA BCS using the string test (see Table 2).

**TABLE 2:** Comparison of mini mental state exam and SA Brief Cognitive Score scores of the literate and illiterate participants with memory impairment.

Variable	Folstein MMSE		SA BCS string	
	Average	range	Average	range
Literate	20.50	10–23	22.50	11–25
Illiterate	18.90	15–22	22.80	19–26

MMSE, mini mental state exam; SA BCS, SA Brief Cognitive Score.

In the illiterate group, the average Folstein MMSE was 18.9, with a mean of 20.5 (range 15–22); and the average score in the SA BCS was 22.8, with a mean 22.55 (range 19–26).

Using the Kruskal–Wallis equality of population rank test, a significant difference (*p* = 0.008) was found between the scores of the illiterate versus the literate patients when the Folstein MMSE was used to assess cognition. When the SA BCS was used, no significant difference was found between the two groups (*p* = 0.79) (*p* < 0.05 taken as significant).

## Discussion

In South Africa, a large number of people are illiterate or have only attended school for a limited number of years. It is estimated that 14.6% of adults (more than 8 million) have difficulty with reading and writing.^14^ Several studies have shown that cognitive performance, as measured by various tests, is influenced significantly by education level.^3,4,5,6,7,15^

Steve Biko Academic Hospital, being a state hospital, caters to a large financially and socially disadvantaged population. In the Neurology Department, there are many patients who are either illiterate or minimally literate who do not perform well on the standard cognitive assessment. Some patients cannot write or draw; therefore, some items have to be omitted when the patient is assessed with the MMSE.

Consequently, the value of using the MMSE in these patients is questionable and cognitive assessment in these individuals is not accurate. It is desirable to have a standardised simple and quick test of cognitive function in patients that have low educational levels, which could be routinely used in a busy neurology outpatient department. In several countries such as China, Bangladesh and India, the original Folstein MMSE has been adapted to accommodate the illiterate and minimally literate population, but no South African studies have been done. Therefore, we aimed to create a South African version of cognitive assessment and evaluate its use in both literate and illiterate individuals.

Our SA BCS was created after careful consideration and comparing the above-mentioned scales as well as consulting with senior neurologists. Our scale was well received by participants who had no memory impairments, both literate and illiterate/minimally literate individuals. It became clear that the illiterate participants improved their scores significantly on the SA BCS when compared to their scores on the Folstein MMSE. On average, healthy participants improved their scores by 6.5 points from the original Folstein MMSE; patients who complained of memory problems and were illiterate/minimally literate improved their scores with an average of almost 4 points. Similar results had been found in the adapted MMSE versions from China, Bangladesh and India.^9,10,11,12^

In our study, patients showed improvement in tests of attention, especially the ‘days of the week backward’ question when compared with spelling ‘WORLD’ backwards or 100 minus 7. Marked improvement was also noted in the illiterate group for questions that require reading and writing skills such as sentence construction instead of writing a sentence; imitating a picture of interlinked fingers instead of obeying a written command; and laying a figure of 8 with a string instead of copying a picture of intersecting pentagons. In total, the SA BCS scores shifted 80% of patients with a score compatible with moderate cognitive impairment on the Folstein MMSE to a category of only mild impairment. One patient improved from the mild cognitive impairment category to normal cognition. This is a very important observation because severity level of cognitive impairment can influence the treatment offered to patients.

In our study, the literate group also showed some improvement in the SA BCS when compared to the Folstein MMSE score. Most of the improvements in the literate group were noted when testing attention where patients scored better in the ‘days of the week backward’ or 20 minus 5 question versus the 100 minus 7 test.

**FIGURE 1:**
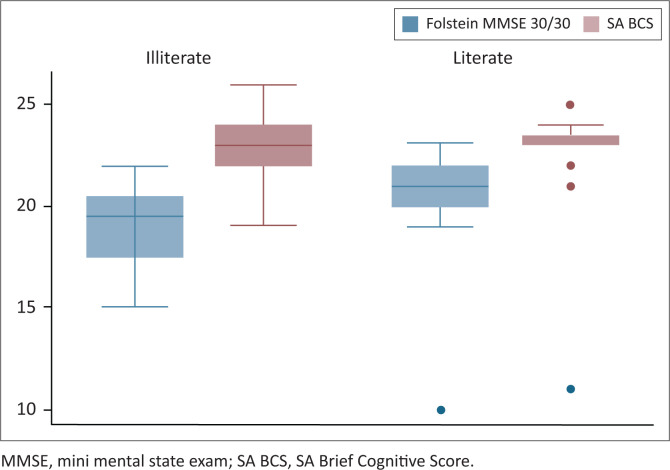
Comparison of the scores for the Folstein versus SA Brief Cognitive Score in both groups of patients.

Our study has several limitations. The study groups consisted of small sample sizes and this could make it difficult for the findings to be extrapolated – ideally, larger numbers of healthy participants and patients with memory impairments need to be tested to confirm the findings. The scale should also be tested on individuals with more severe memory deficits and compared with the original MMSE scores in these patients in the two groups. We had only one patient with severe memory impairment (the outlier on Figure 1, who scored 10 on the MMSE and 11 on the SA BCS). Another limitation of the study was that we used participants with no schooling and those with up to 7 years of education in one group as ‘illiterate/minimally literate’ participants. This could have skewed the results favourably for both the Folstein MMSE and the SA BCS. Ideally, participants with absolutely no schooling should be tested and should also be compared with those with minimal school education. Unfortunately, the participants were also not sex- and age-matched; using age- and sex-matching in future research could contribute to a better follow-up study.

## Conclusion

The SA BCS was well received by illiterate and literate healthy individuals as well as by patients complaining of memory problems. In illiterate individuals, the SA BCS yielded normal scores compared with the Folstein MMSE scores, which would have placed many normal individuals into a cognitively impaired category. In patients with memory problems, the original Folstein MMSE yielded much lower scores in the illiterate patients when compared to literate patients; however, the SA BCS yielded similar scores in both literate and illiterate patients. Illiterate patients’ scores improved significantly when tested with the SA BCS compared with those tested with the Folstein MMSE. Thus, the SA BCS is potentially a useful scale for testing cognition in illiterate or minimally literate individuals in South Africa.

## References

[1] Folstein MF, Folstein SE, McHugh PR. ‘Mini-mental state’ – A practical method for grading the cognitive state of patients for the clinician. J Psych Res. 1975;12(3):189–198. 10.1016/0022-3956(75)90026-6 1202204

[2] Santacruz KS, Swagerty D. Early diagnosis of dementia. Am Fam Physician. 2001;63(4):703–714. 11237085

[3] De Brito-Marques PR, Cabral-Filho JE. The role of education in mini mental state examination: A study done in Northeast Brazil. Arq Neuropsiquiatr. 2004;62(2-A):206–211. 10.1590/S0004-282X2004000200003 15235718

[4] O’Connor DW, Pollitt PA, Treasure FP, Brook CP, Reiss BB. The influence of education, social class and sex on Mini-Mental scores. Psychol Med. 1989;19(3):771–776. 10.1017/S0033291700024375 2798645

[5] Ishizaki J, Meguro K, Ambo H, et al. A normative community-based study of Mini-mental state in elderly adults: The effect of age and educational level. J Gerontol. 1998;53B(6):359–363. 10.1093/geronb/53B.6.P359 9826967

[6] Kochhann R. Evaluation of Mini-Mental State Examination scores according to different age and education in large Brazilian healthy sample. Dementia Neuropsychol. 2009;3(2):88–93. 10.1590/S1980-57642009DN30200004 PMC5619224 29213617

[7] Laks J. Mini-mental state examination norms in a community-dwelling sample of elderly with low schooling in Brazil. Cad Saude Publica Rio de Janeiro. 2007;23(2):315–319. 10.1590/S0102-311X2007000200007 17221080

[8] Aitchison J, Rule P. A quick survey of SADC literacy statistics and projections [homepage on the Internet]. 2006 [cited 2019 Aug 31]. Paper commissioned for the EFA Global monitoring report 2006, Literacy for Life. Centre for Adult Education, University of KwaZulu-Natal, Durban. Available from: https://citeseerx.ist.psu.edu/viewdoc/download?doi=10.1.1.403.7828&rep=rep1&type=pdf

[9] Gelin X, Meyer JS, Huang Y, Du F, Chowdhury M, Quach M. Adapting mini-mental state examination for dementia screening among illiterate or minimally educated elderly Chinese. Int J Geriatr Psychiatry. 2003;18(7):609–616. 10.1002/gps.890 12833305

[10] Kabir ZN, Herlitz A. The Bangla adaptation of mini mental state examination (BAMSE): An instrument to assess cognitive function in illiterate and literate individuals. Int J Geriatr Psychiatry. 2000;15(5):441–450. 10.1002/(SICI)1099-1166(200005)15:5<441::AID-GPS142>3.0.CO;2-O 10822243

[11] Ghose SK, Chowdhury A, Hasan A, et al. Comparison of mini-mental state examination (MMSE) and Bangla Mini-Mental state examination (MMSE-B) among healthy elderly in Bangladesh. J Dhaka Med Coll. 2015;24(1): 30–35. 10.3329/jdmc.v24i1.29559

[12] Ganguli M, Ratcliff G.A. Hindi version of the MMSE: The development of a cognitive screening instrument for a largely illiterate rural elderly population in India. Int J Geriatr Psychiatry. 1995;10(5):367–377. 10.1002/gps.930100505

[13] Ramlall S, Chipps J, Bhigjee AL, Pillay BJ. Screening a heterogeneous elderly South African population for cognitive impairment, the utility and performance of the Mini-Mental state examination, six item screener, subjective memory rating scale and deterioration cognitive observee. Afr J Psychiatry. 2013;16(6):445–455. 10.4314/ajpsy.v16i6.57 24173634

[14] Statistics South Africa. General household survey [homepage on the Internet]. 2016 [cited 2019 Aug 31]. Available from: https://www.statssa.gov.za/publications/P0318/P03182016.pdf

[15] Tripathi R. Age, education and gender effects on neuropsychological functions in healthy Indian older adults. Dement Neuropsychol. 2014;8(2):148–154. 10.1590/S1980-57642014DN82000010 29213896 PMC5619122

